# The impact of brain network microstructural changes on upper limb mirror movements after stroke

**DOI:** 10.3389/fneur.2025.1643870

**Published:** 2025-08-06

**Authors:** Jie Dai, Zhaoqing Li, Jingyan Tao, Shuping Sun, Jianhua Li

**Affiliations:** Department of Rehabilitation Medicine, Sir Run Run Shaw Hospital, Zhejiang University School of Medicine, Hangzhou, China

**Keywords:** stroke, mirror movements, diffusion tensor imaging, surface, electromyography, corpus callosum, corticospinal tract

## Abstract

**Objective:**

This study aimed to investigate the relationship between brain network microstructural changes and surface electromyographic (sEMG) signs of upper limb mirror movements (MMs) in stroke patients, focusing on the corpus callosum and bilateral corticospinal tracts (CSTs).

**Method:**

A retrospective analysis was conducted on 48 stroke patients (mean age 60.63y, range 15-83y) who underwent sEMG and diffusion tensor imaging (DTI). All the stroke patients were in the subacute to chronic phase. sEMG parameters, including overflow percentage (OF) and standardized net excitation (SNE), were measured during unilateral maximum contraction tasks. DTI metrics, such as fractional anisotropy (FA), mean diffusivity (MD), axial diffusivity (AD), and radial diffusivity (RD), were analyzed in the corpus callosum and bilateral CSTs. All DTI metrics were processed and computed using FSL (FSL, version 5.0.5). Correlation analyses were performed between sEMG parameters, DTI metrics, and clinical data.

**Results:**

There was a significant asymmetry in motor overflow between affected and unaffected limbs, with higher OF and SNE in the unaffected limb during affected limb movement. The RCI (right-sided cerebral infarction) group showed more pronounced MMs compared to the LCI (left-sided cerebral infarction) group. The DTI metrics were closely related to age and disease duration. In the LCI group, a negative correlation was found between FA values of the corpus callosum and MMs, and between FA of the CST and MMs in the affected limb.

**Conclusion:**

The study demonstrated that sEMG manifestations of MMs could reflect the motor function of the affected upper limb in stroke patients. Microstructural changes to the corpus callosum and CSTs might be one of the causes of the post-stroke MMs.

## 1 Introduction

Stroke, a leading cause of long-term disability all around the world, is resulting in significant motor deficits, particularly in the upper limbs. These deficits, including hemiparesis and impaired motor control, affect nearly 80% of stroke survivors and significantly hinder their daily activities (1).

Mirror movements (MMs) are involuntary movements that occur in one limb when the voluntary movements are performed with the contralateral limbs (2), which could be seen commonly in the upper limbs after stroke (3–5). It’s showed that the presence and severity of MMs correlated with the degree of motor impairment in the affected limb, suggesting a link to neural reorganization and compensatory mechanisms (6–8). One study shows the compensatory mechanism that the unaffected hemisphere’s motor cortex may facilitate movement in the affected limb through increased interhemispheric connections (7). In another study, Jiang et al. summarized the potential mechanisms underlying mirror movements and proposed that brain infarction might reduce inter-hemispheric inhibition from the lesioned to the intact hemisphere, allowing unintended activity in the resting limb. This indicates a potential compensatory mechanism where the brain attempts to restore motor function by altering the balance of neural activity between the hemispheres (6). A case presentation have demonstrated that MMs are associated with increased activation in the contralesional motor cortex using task-based fMRI (9). In a reported case, a 54-year-old man developed left-hand mirror movements after a chronic right internal-capsule infarct. Task-based fMRI revealed heightened activity in the contralesional motor cortex and ipsilateral cerebellum, supporting the concept of compensatory recruitment (9). Therefore, understanding the relationship between MMs and brain activation patterns may provide valuable insights into the neural mechanisms of motor recovery and may offer a novel treatment to improve upper limb function in stroke survivors (10, 11).

Diffusion Tensor Imaging (DTI) is a non-invasive neuroimaging technique used to assess the microstructure of the white matter tracts of brain. This anisotropy is quantified using metrics such as fractional anisotropy (FA), mean diffusivity (MD), axial diffusivity (AD), and radial diffusivity (RD) (12, 13) to provide detailed information on fiber orientation and structural integrity that is not available through conventional MRI (14). Some previous studies have demonstrated that DTI metrics, such as FA and MD, can predict motor recovery outcomes in stroke patients by assessing the integrity of corticospinal tracts (CSTs) (15–17). Additionally, DTI has been used to track longitudinal changes in white-matter microstructure over 6 months of stroke rehabilitation (18). These applications highlight that DTI is a useful tool in both research and clinical practice in the field of stroke and neurorehabilitation. Since now, there were only a few DTI studies on MMs. One study found that congenital MMs were associated with the abnormal decussation of the CSTs (19). Another study focusing on patients with amyotrophic lateral sclerosis (ALS) revealed that MMs were related to the structural damage of the CSTs, while no significant microstructural changes were observed in the corpus callosum. However, the TMS study in this article revealed that ALS patients had prolonged iSP latency and frequent iSP loss, which meant a functional disturbance in the transcallosal pathways. Therefore, they suggested that functional disturbance of transcallosal pathways might precede microstructural changes in the corpus callosum (20).

The previous studies have showed that sEMG could be used to assess potential mirror movements in neurological disorders, offering a new perspective for early diagnosis and treatment (21, 22). And one study found that sEMG measures of MMs, in the paretic upper limb were positively associated with motor recovery after stroke, particularly in the proximal joints (shoulder and elbow) (7). And in our previous study, we found that during unilateral maximum contraction tasks, the sEMG signs were present in the homologous muscles of both limbs in subacute and chronic stroke patients, with an asymmetry between the two sides (23). Based on the previous hypotheses of MMs, microstructural changes in the corpus callosum and CSTs might be one of the most important pathophysiological mechanisms of MMs after stroke (24). Although the subcortical pathway such as the reticulospinal system and rubrospinal tract may also contribute to this process to some extent (11), considering the difficulty in localization, we will discuss these structures in the future. Therefore, in this article, we aim to explore the correlation between the microstructural changes in the corpus callosum and CSTs and the electromyographic signs of MMs, in order to investigate the possible neurophysiological mechanisms of MMs after stroke.

## 2 Methods

### 2.1 Participants

We reviewed the sEMG records and DTI data conducted at the Rehab Department of Sir Run Run Shaw Hospital from June 2021 to June 2024. Among patients who underwent both sEMG and DTI examinations during hospitalization, those with brain infarction confirmed by magnetic resonance imaging were selected. At last, 48 patients were selected (mean age 60.63y, range 15-83y), who met the following inclusion criteria: Patients hospitalized for ischemic stroke, patients who have completed the sEMG assessment, DTI scan and functional scale assessment, including Brunnstrom Stages of Motor Recovery Scale (BR) and Barthel Index (BI), within 1 week after hospitalization. All the patients were right-handed. And the exclusion criteria were: patients with a history of other diseases affecting the motor function of the upper extremities; patients with poor sEMG data or incomplete clinical data; patients had any limitations in the passive range of motion. According to the lesion location, the patients were divided into two groups: the left-sided cerebral infarction group (LCI, *n* = 24) and the right-sided cerebral infarction group (LCI, *n* = 24). The medical history of the patients was taken from the medical records.

All the patients agreed to use their clinical data and examination results in the study and signed consents during hospitalization. All patient’s identification information was concealed during the study. The experimental protocol was approved by the Ethics committee of Sir Run Run Shaw Hospital, Zhejiang University School of Medicine (2023–0174).

### 2.2 Clinical evaluation

All the patients completed the BI of Activities of Daily Living (ADL) for independence in mobility and personal care (score range: 0–20) and the BR for motor function of affected limb (stages I–VI). These assessments were conducted 1 week after hospital admission and again on the day of discharge. All the clinical evaluations were performed by the same physical therapist, who was blinded to the specific content and purpose of the study, to minimize the potential individual bias.

### 2.3 sEMG

#### 2.3.1 sEMG data acquisition

Patients lay in a height-adjustable bed with legs extended and feet on the bed pedal. Both upper limbs were symmetrically placed alongside the trunk, with elbows fully extended and forearms/wrists in a neutral position. Stabilization straps were applied to the distal forearms to prevent elbow flexion, and a harness secured the trunk to avoid compensatory movements. Bipolar sEMG electrodes (Ag/AgCl) were placed over the biceps brachii muscles bilaterally, with a reference electrode on the left lateral humeral epicondyle. sEMG signals were recorded using a biomedical acquisition system (Model AMT-4) and digitized at 1,000 Hz with a 12-bit A/D converter. High- and low-frequency filters were set at 500 Hz and 20 Hz. Data were collected using custom LabVIEW software and processed offline with Megawin3.1.

For the tasks, participants performed voluntary isometric muscle contractions of the biceps brachii in a fixed order: unilateral contractions on ①the affected side and then ②the unaffected side. They were instructed to contract muscles with maximal force for 5 s, followed by a 5-s rest, three contractions per set. Visual feedback from a root-mean-square (RMS) voltmeter helped maintain steady contractions, with RMS reduction controlled within 10% of the maximum (21). The assessments were conducted by the same physician, blinded to the purpose of study, to minimize individual error.

#### 2.3.2 sEMG data processing

The resting baseline signals was recorded from the resting limb before muscle contraction. RMS values were calculated from the absolute values of sEMG signals in a 3-s segment for both active and resting limbs during two tasks. This 3-s duration was chosen because stroke patients were always difficult to maintain steady force for a long period. The mean RMS values were then determined by averaging the values from three trials for each task. The standardized net excitation (SNE) was defined as the normalized difference between the baseline and irradiated EMG values, reflecting the response of resting muscle to contralateral muscle contraction (7). Additionally, the overflow percentage (OF) was calculated as the ratio of EMG activity in the resting limb to that in the active limb, indicating the overflow of the muscle activation (4).

### 2.4 DTI

#### 2.4.1 MRI protocol

MRI scans were performed using a GE Discovery 750 W 3.0 T MRI system with an 8-channel head coil. The imaging protocols included high-resolution T1-weighted imaging, T2 FLAIR, and DTI. T1-weighted images were acquired using a fast gradient echo sequence with 3D magnetization (TR = 8.5 ms, TE = 3.9 ms, 150 slices, voxel size = 1 × 1 × 1 mm^3^). T2 FLAIR images were obtained with a fast spin-echo sequence (TR = 11,000 ms, TE = 125 ms, 31 slices, voxel size = 0.49 × 0.49 × 6.50 mm^3^). DTI was performed with 32 diffusion directions, a b-value of 1,000 s/mm^2^ (60 slices, voxel size = 1.75 × 1.75 × 1.75 mm^3^, TR = 8,000 ms, TE = 80.7 ms). Radiologists were also blinded to the purpose of the study.

#### 2.4.2 Region of interest selection and calculation of DTI metrics

Based on the existing mechanism hypotheses of MMs, three white matter tracts were selected, including the bilateral CSTs and the commissural fibers of the corpus callosum (23), to investigate the correlation between microstructural damage of white matter tracts and MMs after stroke (9, 25). The microstructural damage was quantified as DTI metrics, including FA, MD, AD (corresponds to *λ*_1_) and RD (corresponds to λ_23_), where λ₁, λ₂, and λ₃ were the first, second, and third eigenvalues of the diffusion tensor (26). All DTI metrics were calculated using FSL (FSL, version 5.0.5).

To obtain the FA, MD, AD and RD values for each white matter tract in each patient, image registration was performed between the MNI152 FA template (FMRIB58 FA) and the FA map of each participant. The MNI152 FA template was registered to the FA map of each participant using FLIRT, followed by nonlinear registration with FNIRT in FSL. Subsequently, the linear registration transformation matrix and the deformation field obtained from the nonlinear registration were saved and applied to the CSTs and corpus callosum regions of interest (ROIs) in the Juelich atlas (27) in MNI152 space to derive the ROIs in each participant’s native space. Additionally, to exclude the influence of cerebrospinal fluid, voxels with MD values greater than 2 μm^2^/ms were removed from the ROIs in the native space.

### 2.5 Statistical analysis

The Chi-square test was used to compare the non-parametric data such as gender between the LCI and RCI groups, while the independent-samples t-test was applied to compare parametric data such as disease duration and age between the two groups. The Mann–Whitney U test was employed to determine differences in sEMG parameters and DTI metrics. To analyze the correlation between sEMG parameters, DTI metrics, and clinical data, the Spearman rank correlation coefficient was used. Given the DTI metrics is more sensitive to age and sex, we used partial correlation adjusting for age and sex and repeat the analysis of WM tracts and clinical measures and sEMG para as additional analysis to check the robustness of the results. Due to the lack of more detailed upper limb function assessment in this study, we divided the patients into Low-Function Group (LFG, *n* = 22, BR ≤ 3) and High-Function Group (HFG, *n* = 26, BR > 3) based on their BR score (22). All the data were presented as Means ± Standard deviation, with the level of significance set at 0.05. Finally, all statistical analyses were completed using SPSS version 24.0 (IBM Corporation, Armonk, NY).

**Figure 1:**
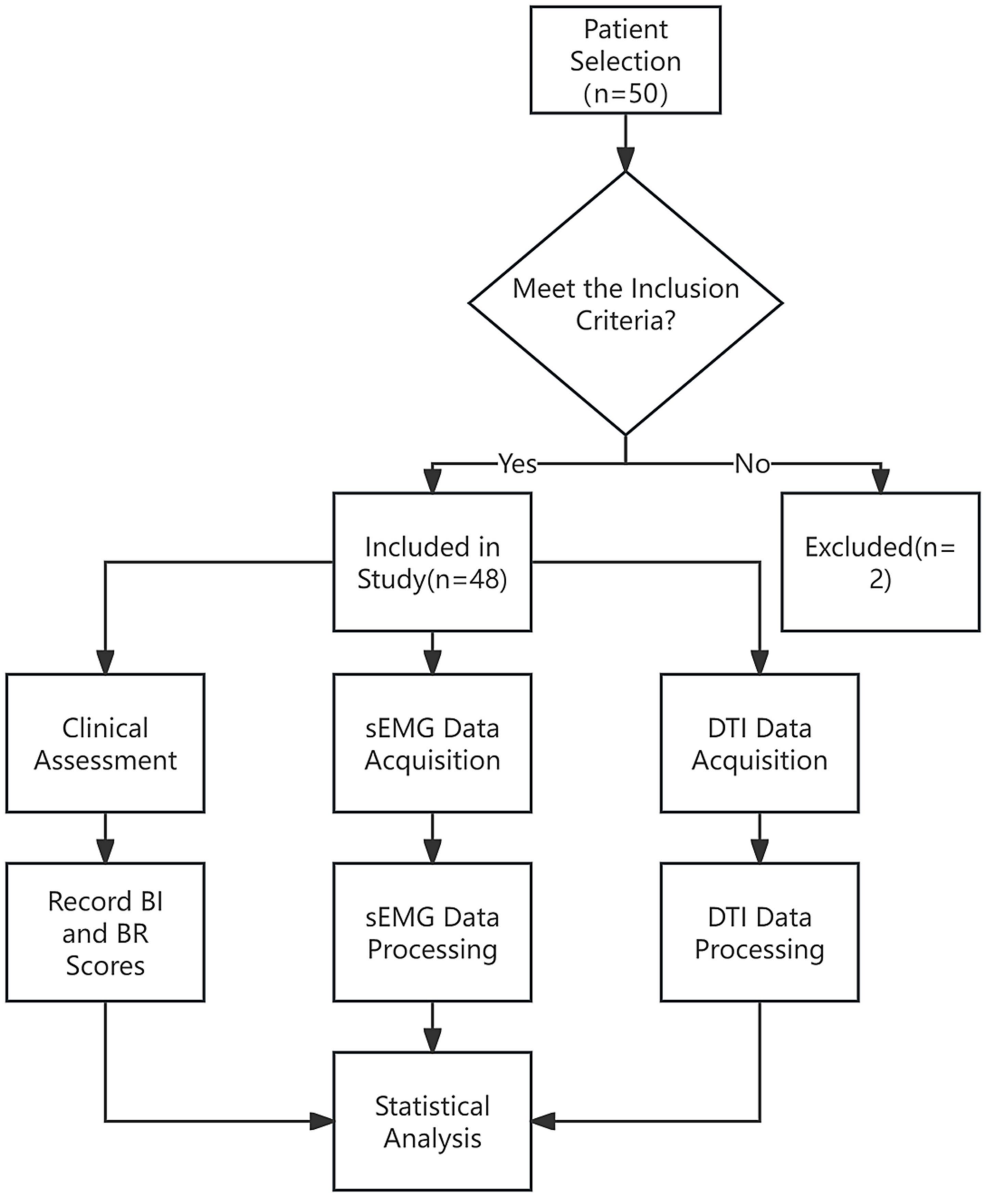
The strobe flow chart of study processing.

The strobe flow chart of this study was showed in Figure 1.

## 3 Results

### 3.1 Participant characteristics

A total of 48 patients were included in this study. The average age, sex ratio, upper limb function grouping, disease duration, and BI scores of the patients are summarized in Table 1. There were no significant differences between the two groups in terms of average age, sex ratio, and BI scores. Although the RCI group had a slightly higher proportion of patients with better upper limb function than the LCI group, the difference was not statistically significant. The disease duration of the RCI group was significantly longer than that of the LCI group (*p* < 0.05).

**Table 1:** Demographics and stroke characteristics.

Demographic characteristics	Stroke patients	LCI group	RCI group
	*n* = 48	*n* = 24	*n* = 24
Age, years (y)	60.63 ± 14.71	58.21 ± 13.98	63.04 ± 15.30
Gender, male: female, *n*	36:12	19:5	17:7
Function classcification, LFG: HFG, n	22:26	14:10	8:16
Disease duration, days (d)	34.10 ± 43.50	26.96 ± 27.53	41.25 ± 54.79*
BI score, *n*	57.23 ± 27.68	53.96 ± 28.63	60.50 ± 26.90

LCI, left-sided cerebral infarction; RCI, left-sided cerebral infarction; HFG, high function group; LFG, low function group; *p < 0.05.

### 3.2 sEMG parameters

**Figure 2:**
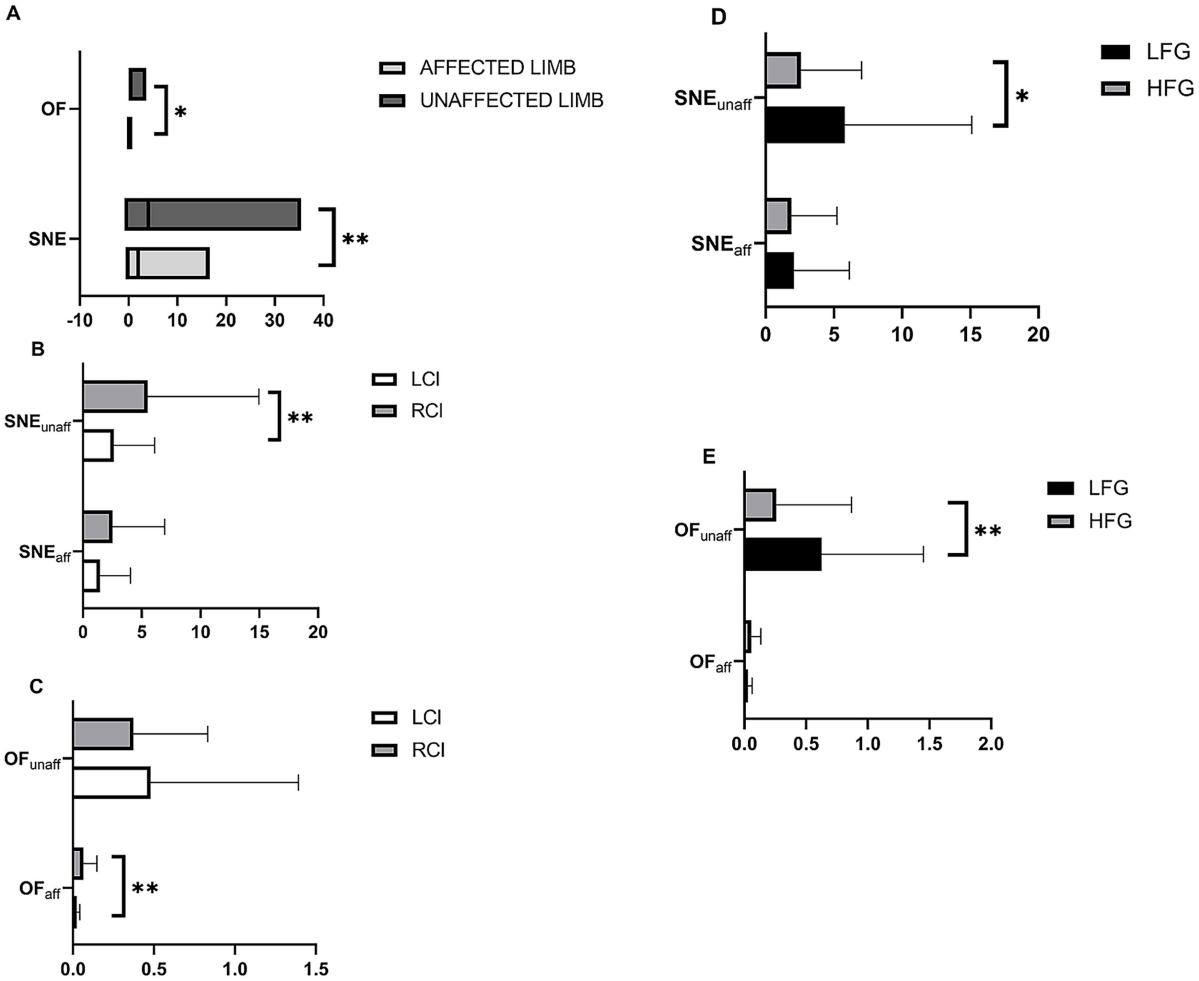
The differences of sEMG parameters **(A)** between affected and unaffected limbs, **(B,C)** between LCI and RCI groups, **(D,E)** between LFG and HFG; *p < 0.05, **p < 0.01.

The differences of sEMG parameters between affected and unaffected limbs were compared, and the results showed in the Figure 2A. And the differences of OF and SNE values between the LCI and RCI groups were compared too, the results showed in the Figures 2B,C. We also compared the differences of those values between the LFG and the HFG. The results were showed in Figures 2D,E.

### 3.3 DTI metrics

**Figure 3:**
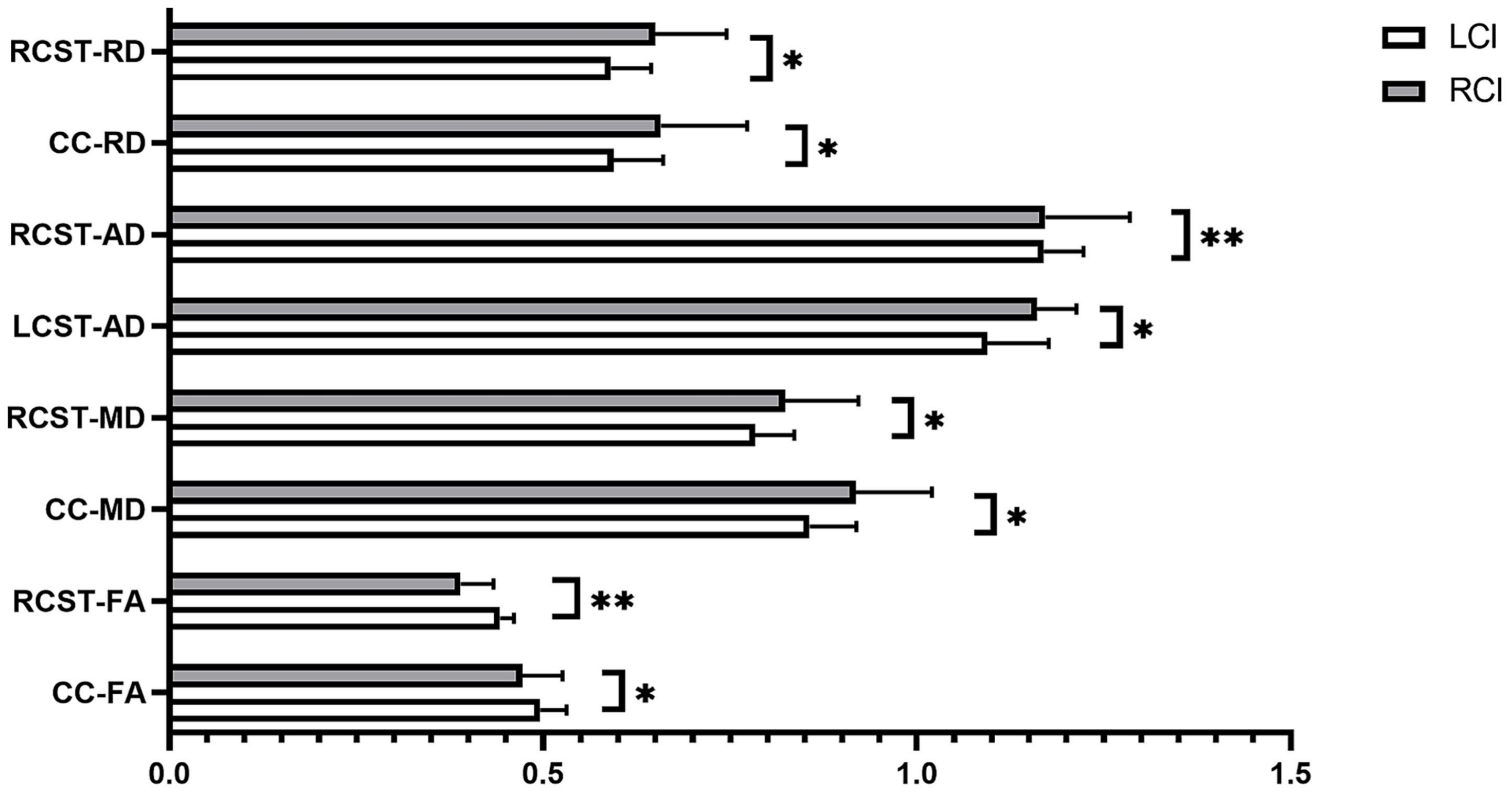
The differences of DTI metrics between LCI and RCI groups; CC-FA, FA value of corpus callosum; RCST-FA, FA value of right corticospinal tract; CC-MD, MD value of corpus callosum; RCST-MD, MD value of right corticospinal tract; LCST-AD, AD value of left corticospinal tract; RCST-AD, AD value of right corticospinal tract; CC-RD, RD value of corpus callosum; RCST-RD, RD value of right corticospinal tract; *p < 0.05; **p < 0.01.

The results of the between-group comparisons of the DTI metrics were showed in Figure 3. No significant statistical differences were found in the DTI metrics between HFG and the LFG.

### 3.4 Correlation analysis

Table 2 show the correlations between age, disease duration time, sEMG parameters, and DTI metrics in all the stroke patients. Among all the stroke patients, the MD, AD, and RD values of the corpus callosum were positively correlated with the age (*p* < 0.01) and disease duration (MD&RD, *p* < 0.01; FA&AD, *p* < 0.05). No significant correlations were found between sEMG parameters and DTI parameters of the corpus callosum. After adjusting for sex and age, the correlation between DTI metrics of the corpus callosum and disease duration became more significant (*p* < 0.01).

**Table 2:** The correlation between clinical data, sEMG parameters and DTI metrics in stroke patients.

	Age, years (y)		Disease duration, days (d)		SNE_aff_		SNE_unaff_		OF_aff_, %		OF_unaff_, %	
	*ρ*	*p*-value	*ρ*	*p*-value	*ρ*	*p*-value	*ρ*	*p*-value	*ρ*	*p*-value	*ρ*	*p*-value
CC-FA	−0.605^**^	0.000	−0.325^*^	0.024	0.051	0.733	−0.125	0.398	0.02	0.891	0.02	0.893
CC-MD	0.638^**^	0.000	0.426^**^	0.003	−0.064	0.665	0.063	0.672	−0.001	0.996	−0.073	0.623
CC-AD	0.568^**^	0.000	0.37^*^	0.01	−0.038	0.799	−0.017	0.91	0.012	0.935	−0.092	0.535
CC-RD	0.649^**^	0.000	0.393^**^	0.006	−0.049	0.741	0.109	0.461	−0.017	0.91	−0.082	0.582
Partial correlation adjusting for age and sex												
CC-FA	–	–	0.559**	0	0.023	0.439	−0.03	0.423	0.112	0.229	0.136	0.184
CC-MD	–	–	0.532**	0	0.052	0.366	0.028	0.426	0.102	0.249	0.044	0.386
CC-AD	–	–	0.381**	0.005	0.089	0.279	0.022	0.443	0.067	0.33	0.046	0.381
CC-RD	–	–	0.563**	0	0.036	0.405	0.03	0.422	0.113	0.228	0.076	0.308

SNE_aff_, SNE value of the affected limb; SNE_unafff_, SNE value of the umaffected limb; OF_aff_, OF value of the affected limb; OF_unafff_, OF value of the umaffected limb; CC-AD, AD value of corpus callosum; ^*^p < 0.05; ^**^p < 0.01.

Table 3 shows the correlations between sEMG parameters, and DTI metrics in the LCI group. we found that the FA (*p* < 0.01), MD (*p* < 0.01), AD (*p* < 0.05), and RD (*p* < 0.05) values of the corpus callosum were also positively correlated with the age of patient. And the disease duration was positively correlated with the AD and RD values of corpus callosum (*p* < 0.05), the AD value of the left CST (*p* < 0.01) and the MD (*p* < 0.05) and RD (*p* < 0.01) value of the right CST. The SNE value of affected limb was negatively correlated with the FA value of corpus callosum and right CST (*p* < 0.05), and was positively correlated with the MD value of left CST (*p* < 0.05). The SNE value of affected limb was positively correlated with the FA value of right CST (*p* < 0.05). However, there wasn’t any correlation between the DTI metrics and sEMG parameters in the RCI group. After adjusting for sex and age, the correlation between DTI metrics of the corpus callosum (FA&MD) and the SNE value of both limbs became more significant(*p* < 0.01) in the LCI group. As expected, there was no correlation between the DTI metrics and sEMG parameters in the RCI group, either.

**Table 3:** The correlation between clinical data, sEMG parameters and DTI metrics in LCI group.

	Age, years (y)		Disease duration, days (d)		SNE_aff_		SNE_unaff_		OF_aff_, %		OF_unaff_, %	
	*ρ*	*p*-value	*ρ*	*p*-value	*ρ*	*p*-value	*ρ*	*p*-value	*ρ*	*p*-value	*ρ*	*p*-value
CC-FA	−0.599**	0.002	−0.166	0.44	−0.429*	0.037	0.186	0.384	−0.112	0.603	0.29	0.169
LCST-FA	−0.051	0.811	−0.093	0.666	−0.068	0.753	0.227	0.286	0.226	0.288	−0.205	0.336
RCST-FA	−0.429*	0.036	−0.199	0.351	−0.426*	0.038	0.443*	0.03	0.249	0.241	0.39	0.06
CC-MD	0.595**	0.002	0.356	0.087	0.318	0.13	−0.27	0.201	0.037	0.862	−0.372	0.073
LCST-MD	0.381	0.066	0.697**	0	0.413*	0.045	−0.236	0.268	0.266	0.209	−0.267	0.207
RCST-MD	0.682**	0	0.24	0.258	0.343	0.101	−0.218	0.306	0.068	0.753	−0.248	0.243
CC-AD	0.451*	0.027	0.414*	0.044	0.183	0.391	−0.256	0.228	0.135	0.528	−0.301	0.153
LCST-AD	0.291	0.168	0.659**	0	0.403	0.051	−0.108	0.616	0.283	0.18	−0.294	0.163
RCST-AD	0.664**	0	0.246	0.247	0.256	0.228	−0.164	0.443	0.186	0.383	−0.153	0.475
CC-RD	0.451*	0.027	0.414*	0.044	0.183	0.391	−0.256	0.228	0.135	0.528	−0.301	0.153
LCST-RD	0.607**	0.002	0.327	0.119	0.377	0.069	−0.241	0.257	0.073	0.736	−0.387	0.062
RCST-RD	0.348	0.096	0.690**	0	0.362	0.082	−0.279	0.187	0.137	0.523	−0.159	0.458
Partial correlation adjusting for age and sex												
CC-FA	–	–	0.265	0.117	0.605**	0.001	0.124	0.291	0.15	0.253	0.527**	0.006
LCST-FA	–	–	0.261	0.12	0.232	0.149	0.406*	0.03	0.28	0.104	−0.03	0.447
RCST-FA	–	–	0.308	0.081	0.439*	0.021	0.53**	0.006	0.309	0.081	0.318	0.075
CC-MD	–	–	0.481*	0.012	0.498**	0.009	0.416*	0.027	0.002	0.497	0.231	0.151
LCST-MD	–	–	0.767**	0	0.374*	0.043	0.133	0.277	0.135	0.275	0.122	0.295
RCST-MD	–	–	0.389*	0.037	0.449*	0.018	0.229	0.153	0.071	0.377	0.197	0.189
CC-AD	–	–	0.484**	0.011	0.279	0.104	0.532**	0.005	0.142	0.264	0.068	0.381
LCST-AD	–	–	0.611**	0.001	0.271	0.112	0.073	0.374	0.283	0.101	0.131	0.28
RCST-AD	–	–	0.309	0.081	0.383*	0.039	0.082	0.358	0.017	0.471	0.155	0.245
CC-RD	–	–	0.484*	0.011	0.279	0.104	0.532**	0.005	0.142	0.264	0.068	0.381
LCST-RD	–	–	0.443*	0.019	0.556**	0.004	0.313	0.078	−0.06	0.396	−0.35	0.055
RCST-RD	–	–	0.747**	0	0.387	0.038	−0.27	0.112	0.006	0.49	0.091	0.344

LCST-FA, FA value of right corticospinal tract; LCST-MD, MD value of right corticospinal tract; LCST-AD, AD value of right corticospinal tract; ^*^p < 0.05; ^**^p < 0.01.

## 4 Discussion

### 4.1 The sEMG parameters of stroke patients

In this study, we found the asymmetry of motor overflow between affected and unaffected limbs again, consistent with our previous research findings (23). We also found the differences between two groups that the RCI group had significantly higher SNE values on the unaffected side and higher OF values on the affected side.

According to our previous research findings, the OF value was closely correlated with the motor function of the affected upper limb (23). Considering that there were more patients with better upper limb function in the RCI group, we sought to verify whether the motor function of the affected upper limb influenced our sEMG results. To this end, we divided the patients into HFG and LFG based on the upper limb BR scores and compared the differences in SNE and OF values between the two groups. The results showed that among all stroke patients, the OF values on the unaffected side were smaller in the HFG than in the LFG, consistent with previous studies (23, 28). We also found that the SNE values on the unaffected side were higher in the HFG than in the LFG. Given the higher number of patients with better upper limb function in the RCI group, this might lead to the higher SNE values on the unaffected limb.

Since the patients had better functional status, the SNE and OF values on the unaffected side would significantly decrease, which meant that as function improves, both SNE and OF values tended to become more symmetrical bilaterally. So we suggest that the OF and SNE values on the unaffected side could, to some extent, reflect the functional status of the affected upper limb in stroke patients and could serve as objective indicators of affected upper limb function, and the OF values might be more sensitive.

It is somewhat special that the OF values on the affected side in the RCI group were higher than that in the LCI group. This phenomenon cannot be explained by the differences in the motor function. It is likely that the difference in the site of lesion might affect the representation of MMs, too. We’ll discuss this below.

### 4.2 The DTI metrics of stroke patients

To further analyze the extent of damage to the white fiber tracts of brain after stroke and its correlation with clinical data. FA, MD, AD, and RD are important metrics used to describe the microstructure of white fibers and the degree of diffusion anisotropy (29, 30).

Our study found significant correlations between age and DTI metrics of both the corpus callosum and bilateral corticospinal tracts (CSTs), consistent with prior research documenting age-related declines in their microstructural integrity and the impact of disease duration on these changes (31–35). Specifically, acute ischemia in the CSTs is associated with tissue edema and cellular swelling, restricting water molecule diffusion and potentially decreasing FA values (36, 37). In the chronic phase, the CSTs may undergo repair and remodeling, with FA values potentially increasing (38). Similarly, the corpus callosum showed worsening damage over time, which may impact motor function recovery of chronic stroke patients (39, 40).

The correlation between post-stroke CST microstructural changes and limb function is more evident. Some studies showed a significant correlation between the motor function of the affected upper limb and FA values in both the ipsilesional and contralesional CSTs in acute stroke patients (41, 42). There are some studies indicating that CSTs lesion load could be used to predict the recovery potential of upper limb function in stroke patients (43, 44). However, our study did not find any differences in DTI metrics of CSTs between the HFG and LFG, which might be due to the relatively small number of cases included in our study. Alternatively, we chose the CST template to define ROIs rather than fiber tracking techniques, and the latter could more accurately determine the extent of the CSTs.

The reorganization of brain architecture following stroke involves both cortical and subcortical changes that contribute to motor recovery. Cortical reorganization is evident through increased activation in the contralesional motor cortex, which may play a compensatory role in motor control, as shown by functional imaging studies and supported by longitudinal research (11, 24, 45). Subcortical pathways, such as the reticulospinal tract, also contribute significantly to motor recovery, particularly in the early stages after stroke, as suggested by studies indicating that mirror movements may originate from the upregulation of these bilaterally organized pathways (9, 11). Longitudinal studies have demonstrated that these changes evolve over time, with mirror movements in the non-paretic hand being exaggerated early after stroke but diminishing as recovery progresses (11). Understanding these mechanisms is crucial for developing targeted rehabilitation strategies to enhance motor recovery in stroke patients.

### 4.3 The correlation between sEMG parameters of mirror movement and DTI metrics

In our study, we found a negative correlation between the FA values of the corpus callosum and the performance of MMs on the affected side, which suggested that the damage to the corpus callosum might lead to the MMs after stroke. The interhemispheric inhibition hypothesis is a generally accepted mechanism of MMs. During unilateral limb movement, the activated cerebral hemisphere can inhibit the activation of the motor cortex in the contralateral hemisphere through the commissural fibers of the corpus callosum. If the corpus callosum damaged, the inhibition might be decreased or disappeared, leading to MMs in the rest limb (24, 46). There are numerous studies supporting this hypothesis. Some studies found that agenesis or partial absence of the corpus callosum was a significant cause of hereditary MMs (47). In patients with unilateral cerebral palsy, the intensity of MMs was correlated with the extent of damage to the corpus callosum. Such damage might impair the ability to control the contralateral motor cortex, and induce MMs (48). According to our findings, the MMs after stroke may have the same mechanism.

Another potential mechanism underlying MMs is the abnormal persistence of the ipsilateral CST (24). The CSTs primarily control voluntary movements of the contralateral limb via crossed fibers. However, brain injury can lead to a reorganization of the projections between the motor cortex and the CSTs, resulting in the persistence of ipsilateral CST from the contralesional motor cortex projecting to the affected limb (49). Consequently, activation of one hemisphere can induce MMs in both limbs (47). In our study, lower FA values in the CST from the contralesional hemisphere were associated with less pronounced MMs in the affected limb, which suggested that the MMs of the affected limb might be induced by the contralesional hemisphere, due to the descending conduction of the ipsilateral CST. We also found a positive correlation between the MD value of the affected CST and MMs in the affected limb, indicating that damage to the CST may exacerbate MMs in that limb. However, the correlations with other metrics were not significant. It was not clear what kind of microstructural changes should be responsible for this findings (30).

After performing partial correlation adjusting for age and sex, we found that the correlation between DTI metrics of the corpus callosum and sEMG parameters became more evident, while the correlation between DTI metrics of the bilateral CSTs and sEMG parameters remained essentially similar to that in the uncontrolled analysis. This finding, on the one hand, confirms the robustness of our analysis. On the other hand, it also reminds us that when analyzing the correlation between DTI metrics of the corpus callosum and other data, it is necessary to control for age and sex variables to ensure the reliability of the results.

Unexpectedly, these correlations only appeared in patients with LCI in our study. We speculate that this may be due to the different roles of the left and right cerebral hemispheres in motor control. It is generally believed that the dominant hemisphere is mainly responsible for fine and complex motor control, such as writing and operating tools. In contrast, the non-dominant hemisphere plays a more important role in spatial perception and coordination during movements (50). Due to the different roles of the two cerebral hemispheres, the mechanisms of MMs after brain damage might also be different. In addition to interhemispheric inhibition and the persistence of abnormal descending pathways, there could be other possible mechanisms of MMs after right-sided cerebral infarction, just as the changes in other brain network micro-structures, such as supplementary motor area (SMA) or premotor cortex (PMC) (24).

This study highlights that sEMG parameters and DTI metrics can serve as valuable tools for assessing motor function and predicting recovery outcomes in stroke patients, potentially enhancing clinical evaluations and guiding personalized rehabilitation strategies. Future research should explore the mechanisms of mirror movements in patients with right-sided cerebral infarction and investigate the dynamic changes in brain microstructure over the course of rehabilitation.

## 5 Limitation

As a retrospective study, several limitations should be noted. Firstly, the absence of a control group prevented us from comparing DTI metrics with those of healthy individuals to identify the abnormal structural changes of the corpus callosum and CSTs in patients with stroke. In future studies, it is essential to establish an appropriate control group. Secondly, the number of patients included in this study was relatively small. The limited sample size may have compromised the stability of the results during intragroup analysis. In future studies, it is necessary to recruit a larger number of eligible participants and, if possible, to restrict the age, functional levels and lesion locations of the subjects to minimize the influences of these variables on the findings.

## 6 Conclusion

In conclusion, it suggested that the sEMG manifestations of MMs after stroke could reflect the motor function and its outcomes of the affected upper limb. And the degeneration of the corpus callosum and bilateral CSTs after stroke were closely related to the age/disease duration. The damage to the corpus callosum and CSTs might be the possible causes of MMs in patients with left-sided cerebral infarction, and there might be other possible mechanisms of MMs in patients with right-sided cerebral infarction.

## Data Availability

The raw data supporting the conclusions of this article will be made available by the authors, without undue reservation.
